# Hypolipidemic Effect of Tomato Juice in Hamsters in High Cholesterol Diet-Induced Hyperlipidemia

**DOI:** 10.3390/nu7125552

**Published:** 2015-12-17

**Authors:** Li-Chen Lee, Li Wei, Wen-Ching Huang, Yi-Ju Hsu, Yi-Ming Chen, Chi-Chang Huang

**Affiliations:** 1 Department of Physical Education, Shih Hsin University, Taipei 11604, Taiwan; lilychen@cc.shu.edu.tw; 2 Department of Neurosurgery, Taipei Medical University-WanFang Hospital, Taipei 11696, Taiwan; nsweili@gmail.com; 3 Graduate Institute of Athletics and Coaching Science, National Taiwan Sport University, Taoyuan 33301, Taiwan; 1010503@ntsu.edu.tw; 4 Graduate Institute of Sports Science, National Taiwan Sport University, Taoyuan 33301, Taiwan; 1041302@ntsu.edu.tw

**Keywords:** tomato juice, hypolipidemic, cholesterol, triglyceride, high-cholesterol diet, lipid-lowering

## Abstract

Tomato is a globally famous food and contains several phytonutrients including lycopene, β-carotene, anthocyanin, and flavonoids. The increased temperature used to produce tomato juice, ketchup, tomato paste and canned tomato enhances the bioactive composition. We aimed to verify the beneficial effects of processed tomato juice from Kagome Ltd. (KOT) on hypolipidemic action in hamsters with hyperlipidemia induced by a 0.2% cholesterol and 10% lard diet (*i.e.*, high-cholesterol diet (HCD)). Male Golden Syrian hamsters were randomly divided into two groups for treatment: normal (*n* = 8), standard diet (control); and experimental (*n* = 32), HCD. The 32 hamsters were further divided into four groups (*n* = 8 per group) to receive vehicle or KOT by oral gavage at 2787, 5573, or 13,934 mg/kg/day for six weeks, designated the HCD-1X, -2X and -5X groups, respectively. The efficacy and safety of KOT supplementation was evaluated by lipid profiles of serum, liver and feces and by clinical biochemistry and histopathology. HCD significantly increased serum levels of total cholesterol (TC), triacylglycerol (TG), high-density lipoprotein cholesterol (HDL-C), and low-density lipoprotein cholesterol (LDL-C), LDL-C/HDL-C ratio, hepatic and fetal TC and TG levels, and degree of fatty liver as compared with controls. KOT supplementation dose-dependently decreased serum TC, TG, LDL-C levels, LDL-C/HDL-C ratio, hepatic TC and TG levels, and fecal TG level. Our study provides experiment-based evidence to support that KOT may be useful in treating or preventing the onset of hyperlipidemia.

## 1. Introduction

Tomato is low in fat and calories, cholesterol free, and a good source of fiber and protein. It is also rich in vitamins A and C, β-carotene, potassium, and lycopene [1]. Tomato is now used in enormous quantities in the fresh state and heads the list of all vegetables as a canned product. Most tomato is consumed as a processed product, such as pastes, concentrates, ketchup, salsa and juice. Processed tomato products are important sources of minerals and vitamins in diets [2]. Tomato and processed tomato contain many health-benefit components, such as lycopene, anthocyanin, ascorbic acid, total phenolics, glycoalkaloids, and tomatine and low levels of carotenoids [3,4,5,6,7,8]. Lycopene bioavailability can be affected by food processing. The bioavailability in food is higher for *cis*-isomers than all-*trans*-isomers. Lycopene bioavailability is higher in processed tomato products than in unprocessed fresh tomatoes [9,10,11].

Tomato juice is made by heating, crushing and simmering tomatoes. To maintain nutrition, tomatoes are usually boiled first to inactivate enzymes that decrease vitamin C and other nutrients when tomatoes are crushed. Some foods lose their nutrient content when they are cooked or juiced, but the heating process actually boosts certain of tomatoes’ properties. Lycopene is more available in tomato juice than in fresh tomatoes because of the heat and oil used to produce juice. Canned and bottled tomato juice is often fortified, thereby increasing the levels of vitamins [12,13]. In a previous study, the US Department of Agriculture recorded that 1/2 cup of tomato juice provides 10% and 35% of the recommended daily amount of vitamins C and A, respectively [14].

Tomato juice is known to have lipid-lowering effects and antioxidant activities [11]. In previous study, a tomato processed product improved blood lipid profiles in postmenopausal hyperlipidemic rats [10]. A high dietary intake of tomato products has atheroprotective effects by significantly reducing liver and serum cholesterol levels [15,16]. Tomato from the processing of tomato products contains many bioactive components, including those that act as antioxidants, such as the vitamins C and E and carotenoids. Lycopene is one of the main carotenoids in tomatoes. Previous study demonstrated that lycopene shows greater stability at low than high temperature and benefits from the processed tomato products. The lycopene bioactivity can be more accurately predicted in processed tomato than fresh tomato because lycopene is more soluble in lipids than water and has greater interaction with cellulose. Therefore, grinding tomato and cooking with oil could increase the bioavailability [17,18,19]. Another study showed that a higher lycopene concentration could protect against cardiovascular disease [20]; tomato-processed foods contain lycopene that can help reduce serum triglyceride (TG) levels with human high fat-induced [21,22,23]. One recent study reported that 13-oxo-9,11-octadecadienoic acid in tomato extract acts as a peroxisome proliferator-activated receptor α (PPARα) agonist and ameliorates obesity-induced dyslipidemia and hepatic steatosis [24].

Many studies used the hamster model to evaluate the hypolipidemic effect because it has many similarities with human fat-induced atherosclerotic disease. Similar to humans, hamsters are endowed with cholesterol ester transfer protein and all of the enzymatic pathways in lipoproteins and bile metabolism; atherosclerotic plaques develop in response to a fat diet in lesion-prone areas similar to humans [25,26,27]. Therefore, we used hamsters to evaluate the preventive effectiveness of supplementation with tomato juice from Kagome Ltd. (KOT) that is produced by a process for increasing lycopene and dietary fiber on hyperlipidemia regulation. We also examined the biochemical parameters and liver tissues by histopathology.

## 2. Experimental Section

### 2.1. Materials, Animals, and Experiment Design

KOT was obtained from Taiwan Kagome Co. (Tainan, Taiwan). In this study, the dose of KOT designed for humans was 22.595 g per day (lyophilized powder), which would be equivalent to a daily recommended dose of KOT at 280 mL/serving/day. To ensure precise and accurate dosing of test animals, KOT was lyophilized by freeze-drying to obtain powder extract. The nutrition facts, dietary fiber and lycopene of KOT were provided by Kagome Co. and are shown in Table 1. The hamster dose (2787 mg/kg) we used was converted from a human-equivalent dose (HED) based on body surface area by the following formula from the US Food and Drug Administration: assuming a human weight of 60 kg, the HED for 22.595 (g)/60 (kg) = 377 × 7.4 = 2787 mg/kg; the conversion coefficient 7.4 was used to account for differences in body surface area between hamsters and human as we recently described [28].

**Table 1:** Nutrition facts of tomato juice from Kagome Ltd. (KOT) (per g lyophilized powder).

Nutrition Facts	Content
	/g KOT (Lyophilized Powder)
Protein	0.12 g
Fat	0
Saturated fat	0
Trans fat	0
Carbohydrate	0.81 g
Sugar	0.53 g
Sodium	1.19 mg
Total calories	3.47 Kcal
Dietary fiber	0.12 g
Lycopene	1.44 mg

From Taiwan Kagome Co. (Tainan, Taiwan).

Specific pathogen-free (SPF) male Golden Syrian hamsters (12 weeks old) were purchased from the National Laboratory Animal Center (NLAC), Taipei City, Taiwan. Animals were housed in the animal facility at National Taiwan Sport University at temperature (22 ± 1 °C) and 50% to 60% relative humidity, with a 12 h light–dark cycle (light on 7:00 a.m.). Distilled water and standard laboratory chow diet (No. 5001; PMI Nutrition International, Brentwood, MO, USA) were provided *ad libitum*. Before the experiments, the hamsters were acclimatized for 1 week to the environment and diet. The Institutional Animal Care and Use Committee (IACUC) of National Taiwan Sport University (NTSU) approved all animal experimental protocols, and the study conformed to the guidelines of the protocol IACUC-10306 approved by the IACUC ethics committee.

**Figure 1:**
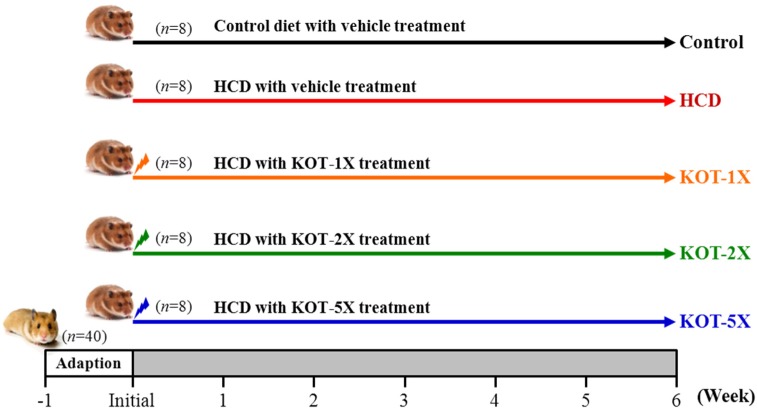
Experimental design for hamsters. Control: standard laboratory diet and the same volume of solution equivalent to body weight (BW). HCD (high-cholesterol diet), HCD and the same volume of solution equivalent to BW; KOT-1X, HCD and 2787 mg/kg/day KOT; KOT-2X, HCD and 5573 mg/kg/day KOT; KOT-5X, HCD and 13,934 mg/kg/day KOT. KOT: tomato juice from Kagome Ltd.

The experimental design is in Figure 1. A total of 40 hamsters were randomly divided into 5 groups for treatment (*n* = 8/each group): (1) control, standard chow diet with vehicle (water); (2) HCD, standard chow (No. 5001) with 0.2% cholesterol and 10% lard diet with vehicle treatment; (3) KOT-1X, HCD with KOT supplementation at 2787 mg/kg; (4) KOT-2X, HCD with KOT supplementation at 5573 mg/kg; (5) KOT-5X, HCD with KOT supplementation at 13,934 mg/kg. The vehicle treatment was the volume of solution to body weight (BW). The food intake and water consumption were monitored daily, and BW was recorded weekly.

### 2.2. HCD Composition

Hamsters were fed a standard chow diet or an HCD adapted from our previous study [29]. The standard chow (No. 5001) contained 3.35 kcal/g with 28.5% as protein, 13.5% as fat and 58.0% as carbohydrates. The HCD contained 0.2% (*wt*/*wt*) cholesterol (Sigma-Aldrich, St. Louis, MO, USA), 10% (*wt*/*wt*) lard (Sigma-Aldrich) and 89.8% (*wt*/*wt*) standard chow, for 3.92 kcal/g with 21.96% as protein, 33.37% as fat and 44.67% as carbohydrates.

### 2.3. Clinical Biochemical Profiles

At the end of the experiment, after 12 h of food deprivation all hamsters were anaesthetized with 5% isoflurane at the rate of 0.5 L/min and euthanized by exsanguination after 12 h of food deprivation. Blood samples were collected from abdominal aortas. Serum was collected by centrifugation at 1500× *g* for 15 min and the clinical biochemical variables including aspartate aminotransferase (AST), alanine aminotransferase (ALT), lactate dehydrogenase (LDH), total protein (TP), blood urea nitrogen (BUN), creatinine, and glucose were measured by using the Beckman DxC 800 analyzer (Beckman Coulter, Brea, CA, USA). Hamsters were sacrificed after 6 weeks of KOT supplementation; liver, kidney, heart and epididymal fat pad (EFP) were removed and tissue weight was recorded for evaluating body composition. All tissue samples were snap-frozen and stored at −80 °C until further analysis.

### 2.4. Liver and Fecal Lipid Analysis

We used a metabolic cage (Muromachi Kikai, Tokyo) to collect hamster feces for analysis of fecal TG and total cholesterol (TC) levels. Fecal lipids were extracted by using chloroform-methanol (2:1, *v*/*v*) with a Bullet Blender (Next Advance, Cambridge, MA, USA). The suspension was filtered through Whatman No. 5 filter paper (Whatman, Maidstone, UK), and the solvent was aspirated, and evaporated. The residue was resuspended in 1 mL of DMSO solution. Fecal TG and TC levels were measured colorimetrically as described previously. Hepatic TG and TC were extracted by chloroform-isopropanol-NP40 (7:11:0.1, *v*/*v*) with a Bullet Blender. After centrifugation (12,000× *g*; 10 min), TG and TC levels were quantified by using a commercial enzymatic kit for TG (No. 10010303) and a kit for TC (No. 10007640) from Cayman Chemical (Ann Arbor, MI, USA).

### 2.5. Histological Staining of Tissues

Liver tissues were carefully removed, minced and fixed in 10% formalin. All samples were embedded in paraffin and cut into 4-μm thick slices for morphological and pathological evaluations. Tissue was stained with hematoxylin and eosin (H&E) and examined under a light microscope equipped with a CCD camera (BX-51, Olympus, Tokyo, Japan) by a veterinary pathologist.

### 2.6. Statistical Analysis

All data are expressed as mean ± SD (standard deviation). Statistical differences were analyzed by one-way analysis of variance (ANOVA) and the Cochran–Armitage test for trend analysis of dose–effect of KOT supplementation with use of SAS 9.0 (SAS Inst., Cary, NC, USA). *p* < 0.05 was considered statistically significant.

## 3. Results and Discussion

### 3.1. Hamster BW and Daily Intake

**Figure 2:**
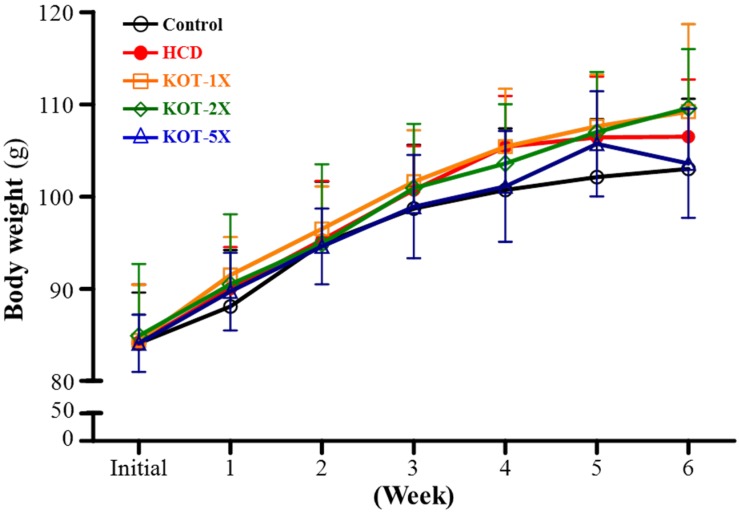
Change in body weight (BW) during the experiment. Golden Syrian hamsters were separated into controls and fed a standard laboratory diet (*n* = 8) or experimental diet (*n* = 32) and treated as in Figure 1. The BW of hamsters was measured once a week in each group. Data are mean ± SD (standard deviation) for *n* = 8 hamster per group. HCD (high-cholesterol diet), HCD and the same volume of solution equivalent to BW; KOT-1X, HCD and 2787 mg/kg/day KOT; KOT-2X, HCD and 5573 mg/kg/day KOT; KOT-5X, HCD and 13,934 mg/kg/day KOT. KOT: tomato juice from Kagome Ltd.

The growth curves for hamsters are in Figure 2. At the start of the experiment, the BW of the five groups did not significantly differ (Table 2). During the experimental period, BW was stable and steadily increased in each group. At the end of the experiment, the BW did not differ among the groups. Therefore, the HCD did not affect BW. With KOT supplementation, the BW curve was still stable and steadily increased, with no significant differences among groups. The daily intake is shown in Table 2. The diet intake did not differ among the groups, but water intake significantly decreased in HCD-induced hyperlipidemia groups (HCD, KOT-1X, -2X and -5X) as compared with controls. This result was same as for our previous study; hamsters fed an HCD to induce hyperlipidemia showed decreased daily water intake [30].

**Table 2:** Body weight (BW) and daily food intake for the experimental groups.

Characteristics	Control	HCD	KOT-1X	KOT-2X	KOT-5X	Trend Analysis
Initial BW (g)	84.1 ± 5.5	84.2 ± 6.3	84.4 ± 6.0	84.9 ± 7.8	84.1 ± 3.1	0.8023
Final BW (g)	103.0 ± 7.6	106.5 ± 6.2	109.2 ± 9.5	109.6 ± 6.4	103.6 ± 5.9	0.6573
Diet intake (g/hamster/day)	8.59 ± 0.57	8.40 ± 0.79	8.31 ± 0.70	8.29 ± 0.73	8.21 ± 0.65	0.2794
Water intake (g/hamster/day)	9.35 ± 1.25 ^c^	8.20 ± 0.80 ^b^	8.13 ± 0.83 ^a,b^	7.67 ± 0.66 ^a,b^	7.25 ± 0.80 ^a^	0.9522

Data are mean ± SD (standard deviation), *n* = 8 hamsters in each group. Control, a standard laboratory diet and orally received the same volume of solution equivalent to BW; HCD (high-cholesterol diet), HCD and the same volume of solution equivalent to BW; KOT-1X, HCD and 2787 mg/kg/day KOT. KOT-2X, HCD and 5573 mg/kg/day KOT. KOT-5X, HCD and 13,934 mg/kg/day KOT. Values in the same row with different superscripts letters (^a–c^) significantly differ at *p* < 0.05 by one-way analysis of variance (ANOVA). HCD (high-cholesterol diet), HCD and the same volume of solution equivalent to BW; KOT-1X, HCD and 2787 mg/kg/day KOT; KOT-2X, HCD and 5573 mg/kg/day KOT; KOT-5X, HCD and 13,934 mg/kg/day KOT. KOT: tomato juice from Kagome Ltd.

### 3.2. Effect of Six-Week KOT Supplementation on Serum Lipid Levels and LDL-C/HDL-C Ratio in Hamsters

**Figure 3:**
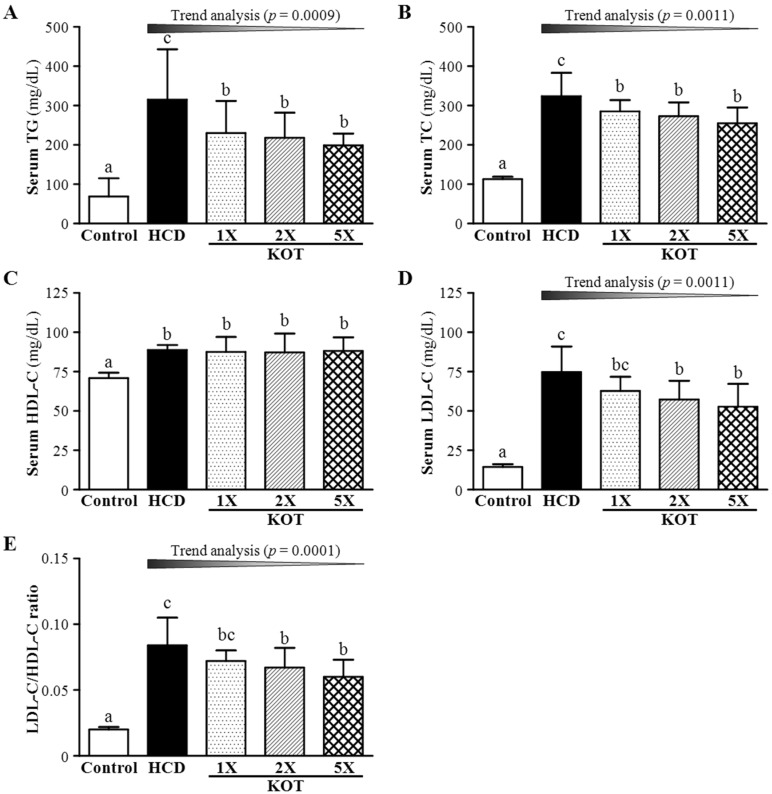
Effect of six-week supplementation with KOT on serum levels of triglycerides (TG) (**A**); total cholesterol (TC) (**B**); high-density lipoprotein cholesterol (HDL-C) (**C**); low-density lipoprotein cholesterol (LDL-C) (**D**); and LDL-C/HDL-C ratio (**E**) in hamsters. Data are mean ± SD (standard deviation), *n* = 8 hamsters in each group. Columns with different letters (a–c) differ significantly at *p* < 0.05 by a one-way ANOVA. HCD (high-cholesterol diet), HCD and the same volume of solution equivalent to BW; KOT-1X, HCD and 2787 mg/kg/day KOT; KOT-2X, HCD and 5573 mg/kg/day KOT; KOT-5X, HCD and 13,934 mg/kg/day KOT. KOT: tomato juice from Kagome Ltd.

At six weeks after KOT supplementation, TG level was 69 ± 46, 315 ± 128, 230 ± 82, 218 ± 64 and 199 ± 30 (mg/dL) in control, HCD, KOT-1X, -2X and -5X groups, respectively (Figure 3A). Hamsters fed an HCD diet showed significantly increased TG level, by 4.59-fold (*p <* 0.0001), as compared with controls. TG level was lower, by 27% (*p =* 0.0354), 30.6% (*p =* 0.0179) and 36.7% (*p =* 0.0053) for KOT-1X, -2X and -5X groups, respectively, than with HCD alone. On trend analysis, serum TG level was dose-dependently decreased with KOT treatment under HCD-induced hyperlipidemia (*p* = 0.0009). TC level was 113 ± 6, 324 ± 59, 285 ± 29 and 255 ± 40 (mg/dL) for control, HCD, KOT-1X, -2X and -5X groups, respectively (Figure 3B), and was higher, by 2.88-fold, with HCD alone than for controls (*p <* 0.0001). TC level was lower, by 12.1% (*p =* 0.0462), 15.6% (*p =* 0.0115) and 21.5% (*p =* 0.0008) for KOT-1X, -2X and -5X groups, respectively, than with HCD alone. On trend analysis, TG level was dose-dependently decreased with the KOT treatment (*p* = 0.0011). Therefore, our HCD diet model could induce hyperlipidemia in healthy hamsters. Furthermore, KOT could reduce serum TC and TG levels under HCD-induced hyperlipidemia.

After six-week KOT supplementation, HDL-C level was 70 ± 3.4, 88.8 ± 3.1, 87.5 ± 9.5, 87.2 ± 12.0 and 88.2 ± 8.6 (mg/dL) for control, HCD, KOT-1X, -2X and -5X groups, respectively (Figure 3C), and was significantly higher, by 1.25-fold (*p <* 0.0001), 1.23-fold (*p =* 0.0002), 1.23-fold (*p =* 0.0003) and 1.24-fold (*p =* 0.0001) for HCD, KOT-1X, -2X and -5X groups, respectively, than controls but did not differ among HCD, KOT-1X, -2X and -5X groups. Thus, HCD diet could significantly increase HDL-C level in healthy hamsters. LDL-C level was 14.4 ± 1.8, 74.7 ± 16.3, 62.8 ± 8.9 and 57.3 ± 11.9 and 52.8 ± 14.4 (mg/dL) for control, HCD, KOT-1X, -2X and -5X groups, respectively (Figure 3D), and was higher, by 5.19-fold (*p <* 0.0001), with HCD alone than for controls. In addition, LDL-C level was lower, by 15.9% (*p =* 0.0517), 23.4% (*p =* 0.0055) and 29.4% (*p =* 0.0007) for KOT-1X, -2X and -5X groups, respectively, than with HCD alone. On trend analysis, KOT supplementation dose-dependently decreased LDL-C level under HCD-induced hyperlipidemia (*p =* 0.0011). Thus, KOT supplementation could inhibit increased HDL-C level under HCD-induced hyperlipidemia.

LDL-C is generated by lipolysis of VLDL-C. The function of LDL-C is to deliver cholesterol to cells, where it is used in membranes or for the synthesis of steroid hormones [31,32,33]. Additionally, increased concentrations of LDL-C are strongly associated with increased atherosclerosis, because LDL becoming oxidized LDL inhibits macrophage migration and promotes lipid uptake by macrophages, which become foam cells that accumulate in atherosclerotic plaques [34,35]. Thus, the level of LDL-C is linked to atherosclerosis-related cardiovascular consequences [36,37]. In our study, KOT could have modulatory effects on LDL-C levels and show health benefits with specific ingredients that have a pharmacological effect on hyperlipidemia. A previous study demonstrated that tomatine, a major component of tomato, decreased serum LDL-C level but had no effect on HDL-C level in hamsters fed an HCD [38]. This result was similar to our data.

The ratio of LDL-C/HDL-C is a criterion for evaluating the efficiency of cholesterol-lowering capacity. If the ratio is low, atherosclerotic risk factors are decreased [39,40]. The ratio of LDL-C/HDL-C was 0.20 ± 0.02, 0.84 ± 0.21, 0.72 ± 0.08, 0.67 ± 0.15 and 0.60 ± 0.13 for control, HCD, KOT-1X, -2X and -5X groups, respectively (Figure 3E), and was higher, by 3.54-fold (*p <* 0.001), with HCD alone than for controls. Moreover, LDL-C/HDL-C ratio was lower, by 14.9% (*p =* 0.0727), 21.2% (*p =* 0.0113), 29.5% (*p =* 0.0007), for KOT-1X, -2X and -5X groups, respectively, than with HCD alone. On trend analysis, the ratio of LDL-C/HDL-C was dose-dependently decreased with KOT supplementation under HCD-induced hyperlipidemia (*p =* 0.0001).

### 3.3. Effect of Six-Week KOT Supplementation on Hepatic TG and TC Levels in Hyperlipidemic Hamsters

**Figure 4:**
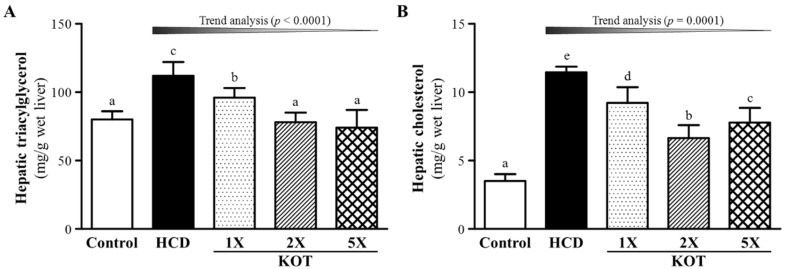
Effect of a six-week supplementation with KOT on hepatic triglycerides (TG) (**A**) and total cholesterol (TC) (**B**) levels in hyperlipidemic hamsters. Data are the mean ± SD, *n* = 8 hamsters in each group. Columns with different letters (a, b, c, and d) significantly differ at *p* < 0.05 by a one-way ANOVA. HCD (high-cholesterol diet), HCD and the same volume of solution equivalent to BW; KOT-1X, HCD and 2787 mg/kg/day KOT; KOT-2X, HCD and 5573 mg/kg/day KOT; KOT-5X, HCD and 13,934 mg/kg/day KOT. KOT: tomato juice from Kagome Ltd.

At the end of the experiment, liver TG content was 80.6 ± 6, 112 ± 10, 96 ± 7, 78 ± 7 and 74 ± 13 (mg/g liver) for control, HCD, KOT-1X, -2X and -5X groups, respectively (Figure 4A), and was higher, by 1.40-fold (*p* < 0.001), with HCD alone than for controls. Liver TG content was lower, by 14.8% (*p =* 0.0007), 30.2% (*p <* 0.0001) and 33.8% (*p <* 0.0001) for KOT-1X, -2X and -5X groups, respectively, than with HCD alone. On trend analysis, liver TG content was dose-dependently decreased with KOT supplementation under HCD-induced hyperlipidemia (*p* < 0.0001). Furthermore, liver TC content was 3.51 ± 0.49, 11.46 ± 0.41, 9.22 ± 1.15, 6.64 ± 0.96 and 7.78 ± 1.07 (mg/g liver) for control, HCD, KOT-1X, -2X and -5X groups, respectively (Figure 4B), and was higher, by 3.27-fold (*p* < 0.001), with HCD alone than for controls. Liver TC content was lower, by 19.6% (*p <* 0.0001), 42.0% (*p <* 0.0001) and 32.1% (*p <* 0.0001) for KOT-1X, -2X and -5X groups, respectively, than with HCD alone. On trend analysis, liver TC content was dose-dependently decreased with KOT supplementation (*p =* 0.0001). Accumulation of liver fat is often associated with abnormal accumulation of TGs in liver [41]. Therefore, KOT supplementation could significantly mitigate the increased liver TC and TG content induced by the HCD hyperlipidemia model. One recent study demonstrated that 9-oxo-10(E),12(Z),15(Z)-octadecatrienoic acid in tomato extract promotes fatty acid metabolism via PPARα activation in liver cells and has potential for use in the management of dyslipidemia [42]. In addition, 13-oxo-9(Z),11(E),15(Z)-octadecatrienoic acid in the extract of tomato induced PPARγ expression in adipose tissue and resulted in the regulation of adipogenesis [43]. From these previous studies, we suggest that KOT may activate PPARα and PPARγ by two different pathways to reduce TG level and increase insulin sensitization; there were activation of PPARα reduces triglyceride level and is involved in regulation of energy homeostasis and activation of PPARγ causes insulin sensitization and enhances glucose and enhances fatty acids metabolism.

### 3.4. Effect of Six-Week Supplementation with KOT on Fecal TG and TC Levels in Hyperlipidemic Hamsters

**Figure 5:**
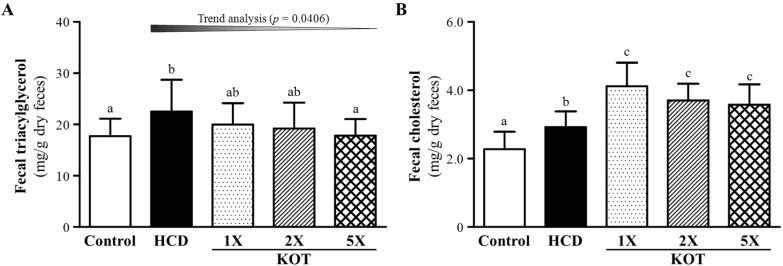
Effect of six-week KOT supplementation on fecal triglycerides (TG) (**A**) and total cholesterol (TC) (**B**) levels in hyperlipidemic hamsters. Data are mean ± SD, *n* = 8 hamsters in each group. Columns with different letters (a–c) significantly differ at *p* < 0.05 by a one-way ANOVA. HCD (high-cholesterol diet), HCD and the same volume of solution equivalent to BW; KOT-1X, HCD and 2787 mg/kg/day KOT; KOT-2X, HCD and 5573 mg/kg/day KOT; KOT-5X, HCD and 13,934 mg/kg/day KOT. KOT: tomato juice from Kagome Ltd.

After the six-week KOT supplementation, we collected all hamsters’ feces for analysis of fecal TG and TC levels. At the end of the experiment, the fecal TG content was 17.7 ± 3.4, 22.5 ± 6.2, 20.0 ± 4.2, 19.2 ± 5.0 and 17.8 ± 3.2 (mg/g feces) in control, HCD, KOT-1X, KOT-2X and KOT-5X, respectively (Figure 5A). The fecal TG content of the HCD group was significantly higher, by 1.27-fold (*p =* 0.0415), as compared with the control. The fecal TG content was decreased by 20.9% (*p =* 0.044) with KOT-5X than with HCD alone. On trend analysis, KOT supplementation dose-dependently decreased fecal TG content under HCD-induced hyperlipidemia (*p =* 0.0406). Fecal TC content was 2.28 ± 0.51, 2.92 ± 0.46, 4.12 ± 0.69, 3.70 ± 0.49 and 3.58 ± 0.59 (mg/g feces) for control, HCD, KOT-1X, -2X and -5X groups, respectively (Figure 5B), and was higher, by 1.28-fold (*p =* 0.0273), with HCD alone than for controls. Furthermore, fecal TC content was higher, by 1.41-fold (*p* = 0.0001), 1.27-fold (*p* = 0.0078) and 1.23-fold (*p* = 0.0224), for KOT-1X, -2X and -5X groups, respectively, than with HCD alone. Therefore, our HCD could increase both fecal TG and TC content. KOT treatment could reduce excessive fecal TG levels and increase fecal TC level excretion. A previous study demonstrated that tomatine decreased serum LDL-C level via formation of a tomatine-cholesterol complex, which was subsequently excreted in feces. Alternatively, the TG and TC content may be reduced by a diet rich in fiber, which may reduce the risk of cardiovascular disease by several mechanisms. Many studies showed significantly reduced cholesterol level associated with dietary fiber intake and cholesterol exerted by feces [44]. We previously showed that viscous flaxseed dietary fibers may be useful for lowering blood cholesterol level than fibers in the solid state [45]. KOT, in accordance with the intervention, allows for decreasing intestinal absorption of dietary lipids and also affects cholesterol homeostasis and lipid transport in the gut, which could explain the decreased hepatic and fecal TG levels with KOT supplementation.

### 3.5. Effect of KOT Supplementation on Tissue Weight at the End of the Experiment

Weight of extracted kidney and hearts and relative kidney and heart weight (%) did not differ among groups (Table 3). The liver weight was 2.92 ± 0.21, 4.52 ± 0.62, 4.22 ± 0.36, 4.09 ± 0.32, 3.89 ± 0.26 (g) for control, HCD, KOT-1X, -2X, -5X groups, respectively. The liver weight was higher, by 1.55-fold (*p <* 0.0001), with HCD than for controls. Additionally, liver weight was lower, by 9.5% (*p =* 0.0295) and 13.9% (*p =* 0.0022), for KOT-2X and -5X groups, respectively, than with HCD alone. On trend analysis, liver weight was dose-dependently decreased with KOT supplementation under HCD-induced hyperlipidemia (*p =* 0.0064). In contrast, epididymal fat pad (EFP) weight was 1.75 ± 0.23, 2.42 ± 0.55, 2.04 ± 0.23, 1.94 ± 0.20 and 1.78 ± 0.17 (g) for control, HCD, KOT-1X, -2X, -5X groups, respectively, and was higher, by 1.38-fold (*p =* 0.0001), with HCD than for controls. The EFP weight was lower, by 15.6% (*p =* 0.0202), 19.8% (*p =* 0.0040) and 26.4% (*p =* 0.0002), for KOT-1X, -2X and -5X group, respectively, than with HCD alone and did not differ among control, KOT-1X, -2X and -5X groups. Thus, KOT supplementation could decrease the body fat content of hamsters. In addition, relative EFP weight was 1.71% ± 0.27%, 2.26% ± 0.48%, 1.88% ± 0.29%, 1.77% ± 0.14% and 1.71% ± 0.01% for control, HCD, KOT-1X, -2X and -5X groups, respectively, and was higher (*p =* 0.0005), by 1.32-fold, with HCD alone than for controls. The relative EFP weight was lower, by 17.0% (*p =* 0.0117), 22.0% (*p =* 0.0015) and 24.4% (*p =* 0.0005), for KOT-1X, -2X and -5X groups, respectively, than with HCD alone. On trend analysis, KOT supplementation dose-dependently decreased EFP weight and relative EFP under HCD-induced hyperlipidemia (*p <* 0.0001 and *p =* 0.0004, respectively). Above all, HCD-induced hyperlipidemia could increase liver and EFP weight and relative weight. Furthermore, KOT supplementation could prevent the adipose tissue accumulation induced by an HCD in hyperlipidemic hamsters.

**Table 3:** Tissue weights at the end of the experiment.

Organ Weight	Control	HCD	KOT-1X	KOT-2X	KOT-5X	Trend Analysis
Liver (g)	2.92 ± 0.21 ^a^	4.52 ± 0.62 ^c^	4.22 ± 0.36 ^b,c^	4.09 ± 0.32 ^b^	3.89 ± 0.26 ^b^	0.0004
Kidney (g)	1.00 ± 0.06	1.01 ± 0.06	1.04 ± 0.07	1.01 ± 0.05	0.99 ± 0.04	0.3862
Heart (g)	0.48 ± 0.05	0.50 ± 0.04	0.47 ± 0.04	0.46 ± 0.03	0.49 ± 0.05	0.1733
EFP (g)	1.75 ± 0.23 ^a^	2.42 ± 0.55 ^b^	2.04 ± 0.23 ^a^	1.94 ± 0.20 ^a^	1.78 ± 0.17 ^a^	<0.0001
Relative liver (%)	2.85 ± 0.32 ^a^	4.25 ± 0.52 ^c^	3.89 ± 0.49 ^b,c^	3.74 ± 0.32 ^b^	3.76 ± 0.24 ^b^	0.0172
Relative kidney (%)	0.98 ± 0.12	0.95 ± 0.08	0.96 ± 0.08	0.92 ± 0.08	0.95 ± 0.03	0.8067
Relative heart (%)	0.47 ± 0.06	0.47 ± 0.05	0.44 ± 0.05	0.43 ± 0.05	0.47 ± 0.03	0.9285
Relative EFP (%)	1.71 ± 0.27 ^a^	2.26 ± 0.48 ^b^	1.88 ± 0.29 ^a^	1.77 ± 0.14 ^a^	1.71 ± 0.09 ^a^	0.0004

Data are mean ± SD (standard deviation), *n* = 8 hamsters per group. Values in the same row with different superscripts letters (^a–c^) significantly differ at *p* < 0.05 by one-way analysis of variance (ANOVA); EFP: Epididymal fat pad. HCD (high-cholesterol diet), HCD and the same volume of solution equivalent to BW; KOT-1X, HCD and 2787 mg/kg/day KOT; KOT-2X, HCD and 5573 mg/kg/day KOT; KOT-5X, HCD and 13,934 mg/kg/day KOT. KOT: tomato juice from Kagome Ltd.

### 3.6. Effect of KOT Supplementation on Biochemical Analyses at the End of the Experiment

**Figure 6:**
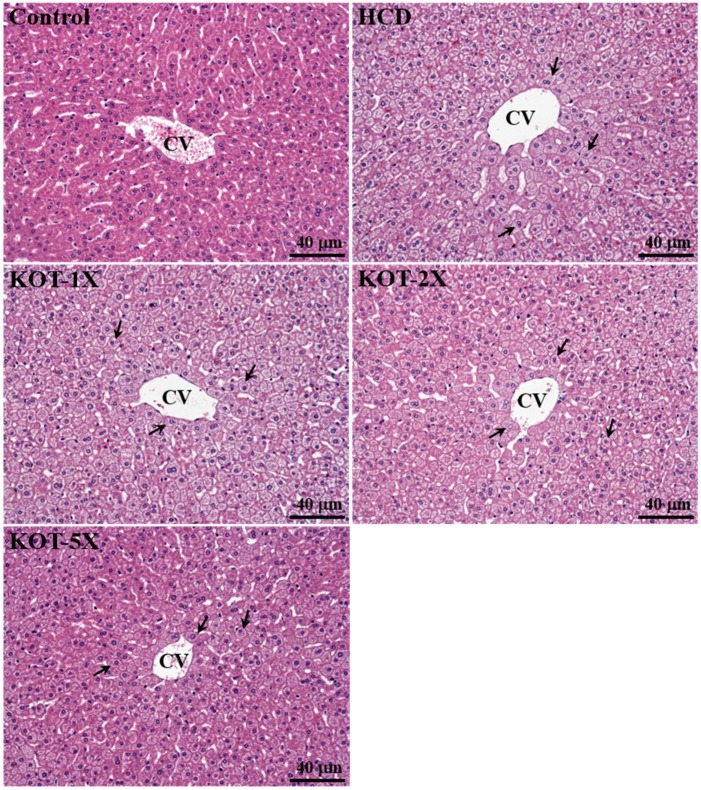
Effect of six-week KOT supplementation on morphology of liver tissues in hyperlipidemic hamsters. Arrows indicate fat droplets. Specimens were photographed by light microscopy. CV, central vein. (H & E stain, magnification: ×200, Scale bar, 40 μm). HCD (high-cholesterol diet), HCD and the same volume of solution equivalent to BW; KOT-1X, HCD and 2787 mg/kg/day KOT; KOT-2X, HCD and 5573 mg/kg/day KOT; KOT-5X, HCD and 13,934 mg/kg/day KOT. KOT: tomato juice from Kagome Ltd.

In our study, we observed the beneficial effects of KOT on indicators of lipid-lowering capacity. We further investigated whether six-week KOT treatment had any adverse effect on other biochemical markers in hamsters. We examined the tissue- and health status-related biochemical parameters and liver tissues by histopathology (Table 4 and Figure 6). KOT supplementation for six weeks had no adverse effects. Levels of biochemical indices, including albumin, total protein (TP), blood urea nitrogen (BUN), creatinine and glucose, did not differ among groups (*p* > 0.05, Table 4). Serum aspartate aminotransferase (AST) and alanine aminotransferase (ALT) activity was higher, by 1.29-fold (*p* = 0.0183) and 1.15-fold (*p* = 0.0224), respectively, with HCD alone than for controls. In addition, KOT supplementation under HCD-induced hyperlipidemia could significantly decrease the serum AST, ALT and LDH activity, respectively, as compared with HCD alone. On trend analysis, serum AST and ALT levels were dose-dependently decreased (*p* = 0.0031, *p* = 0.0006) under HCD-induced hyperlipidemia with KOT supplementation. Therefore, the effect of KOT on decreasing AST and ALT activity was associated with decreased adipose tissue accumulation. The most important adverse side effects of statins (cholesterol-lowering drugs) are increased concentration of liver enzymes and muscle problems [46]. As compared to statins, KOT did not cause liver damage. Therefore, KOT supplementation could provide alternative nutrient supplementation to ameliorate the side effects of statins and has a potential effect on lowering hyperlipidemia.

**Table 4:** Biochemical analysis of KOT treatment groups at the end of experiment.

Parameters	Control	HCD	KOT-1X	KOT-2X	KOT-5X	Trend Analysis
AST (U/L)	48 ± 7 ^a^	62 ± 23 ^b^	37 ± 4 ^a^	38 ± 4 ^a^	37 ± 6 ^a^	0.0031
ALT (U/L)	69 ± 9 ^a^	80 ± 13 ^b^	63 ± 4 ^a^	61 ± 7 ^a^	61 ± 7 ^a^	0.0006
LDH (U/L)	151 ± 22 ^a,b^	155 ± 38 ^b^	151 ± 31 ^a,b^	145 ± 21 ^a,b^	127 ± 15 ^a^	0.0689
Albumin (g/dL)	3.2 ± 0.1	3.3 ± 0.1	3.3 ± 0.1	3.2 ± 0.1	3.2 ± 0.1	0.2660
TP (g/dL)	5.3 ± 0.1	5.4 ± 0.2	5.5 ± 0.2	5.4 ± 0.1	5.4 ± 0.1	0.7738
BUN (mg/dL)	18.3 ± 1.2	17.9 ± 1.5	17.5 ± 1.4	17.3 ± 1.4	17.4 ± 2.1	0.4757
Creatinine (mg/dL)	0.16 ± 0.03	0.17 ± 0.05	0.18 ± 0.01	0.16 ± 0.03	0.18 ± 0.02	0.9734
Glucose (mg/dL)	157 ± 28	157 ± 28	162 ± 39	158 ± 41	168 ± 28	0.4517

Data are mean ± SD for *n* = 8 hamsters per group. Values in the same row with different superscripts letters (^a–c^) significantly differ at *p* < 0.05 by one-way analysis of variance (ANOVA). AST, aspartate aminotransferase; ALT, alanine aminotransferase; LDH, lactate dehydrogenase; TP, total protein; BUN, blood urea nitrogen. HCD (high-cholesterol diet), HCD and the same volume of solution equivalent to BW; KOT-1X, HCD and 2787 mg/kg/day KOT; KOT-2X, HCD and 5573 mg/kg/day KOT; KOT-5X, HCD and 13,934 mg/kg/day KOT. KOT: tomato juice from Kagome Ltd.

### 3.7. Effect of KOT Supplementation on Histology at the End of the Experiment

In a previous study, the high-fat diet-induced pathological morphology of livers significantly differed among rodent species; the fat was microvesicular in hamsters and mixed (macro- and microvesicular) in mice [47]. Liver slices from our hamsters fed a normal chow diet showed a clear hepatic cord and sinusoid (Figure 6). Significant fatty steatosis was detected in all animals of the HCD, KOT-1X, -2X and -5X groups, with hepatocytes comprising microvesicles filled with small lipid droplets, which is similar to the previous pathological observation. The degree of fatty steatosis was significantly lower in the KOT-5X than other HCD-induced hyperlipidemic groups.

## 4. Conclusions

In our study, KOT had lipid-lowering actions by decreasing serum TG and TC levels, liver TG and TC levels, fecal TG levels and serum LDL-C and LDL-C/HDL-C levels in hyperlipidemic hamsters. Six-week KOT supplementation significantly improved the hyperlipidemia syndrome in hamsters. Hamsters showed decreased cholesterol levels in serum, and the KOT effect was exerted via increased fecal lipid excretion. In biochemical study, we found no gross abnormalities attributed to KOT treatment. Thus KOT may be beneficial to human health by reducing the risk of developing cardiovascular disease. Previous studies have demonstrated that the tomato glycoalkaloid tomatine lowered serum LDL-C and cholesterol levels in hamsters and mice [38,48]. The possible mechanism for reducing serum cholesterol level by KOT is inhibition of acyl-CoA:cholesterol acyl-transferase (ACAT)-1 and (ACAT)-2; ACAT-1 is located in the Kupffer cells of the liver, kidneys, and adrenal cortical cells, an important component of cellular cholesterol homeostasis [49]. We provide experiment-based evidence to support that KOT may have potential as a therapy for reducing blood lipid levels and lowering hyperlipidemic effects.
